# Extracellular Vesicles (EVs) Derived from Mesenchymal Stem Cells (MSCs) as Adjuvants in the Treatment of Chronic Kidney Disease (CKD)

**DOI:** 10.3390/cells14060434

**Published:** 2025-03-14

**Authors:** Paloma Noda, Ana L. R. Francini, Flavio Teles, Samuel J. Júnior, Fernando L. A. Fonseca, Fernanda T. Borges, Adão C. Sobrinho, Noemi Taniwaki, Irene L. Noronha, Camilla Fanelli

**Affiliations:** 1 Laboratory of Cellular, Genetic and Molecular Nephrology, Renal Division, Faculty of Medicine, University of São Paulo, Av. Dr. Arnaldo, 455, 4° Andar, 4304, São Paulo 01246-903, SP, Brazilirenenor@usp.br (I.L.N.); 2 Renal Division, Faculty of Medicine, Federal University of Alagoas, Maceio 57200-000, AL, Brazil; 3 Department of Clinical Laboratory, University Center of ABC Medical School, Santo Andre 09060-650, SP, Brazil; 4 Departament of Medicine, Nephrology Division, Paulista School of Medicine, Federal University of São Paulo, São Paulo 04023-062, SP, Brazil; fernanda.teixeiraborges@gmail.com; 5 Laboratory of Cellular Biology, Department of Pathology, School of Medicine, University of São Paulo, São Paulo 01246-903, SP, Brazil; 6 Laboratory of Electron Microscopy, Instituto Adolfo Lutz, São Paulo 01246-000, SP, Brazil

**Keywords:** extracellular vesicles, chronic kidney disease, cell therapy, mesenchymal stem cells

## Abstract

Chronic kidney disease (CKD) is considered an important health issue worldwide. The renin–angiotensin–aldosterone system (RAAS) blockade through the administration of angiotensin II receptor blockers, such as Losartan (LOS), has been considered the best strategy for CKD treatment for decades. However, this approach promotes only partial detention of CKD progression and cannot reverse renal damage. The aim of the present study was to investigate whether the therapeutic administration of extracellular vesicles (EVs) derived from adipose stem cells (ASCs), associated to LOS treatment, would promote additional renoprotection in rats underwent the 5/6 renal ablation CKD model. ASC-derived EV were administered locally, in the renal subcapsular area, 15 days after CKD induction, when LOS therapy also began. Animals were followed for additional 15 days and our results demonstrated that subcapsular injection of ASC-derived EV associated with LOS significantly reduced glomerulosclerosis, renal interstitial infiltration by myofibroblasts, and macrophages in the 5/6 CKD model. Additionally, LOS + EV abrogated systemic hypertension, proteinuria, and albuminuria, and stimulated local gene overexpression of the endogenous anti-inflammatory *Il-4*. Although more studies are still required to establish the best EV dose and administration route, these findings point to therapy with ASC-derived EV as a potential adjuvant in CKD treatment

## 1. Introduction

Chronic kidney disease (CKD) is an incurable and very debilitating condition that leads to progressive loss of renal function and the need for renal replacement therapy (RRT). The number of patients currently affected by CKD in the world exceeds 850 million, which represents more than 10% of the global population. Due to its high morbidity and mortality, and in face of the high costs of its treatment, CKD is considered an important public health problem worldwide, motivating the scientific community to search for innovative and more effective therapeutic strategies to detain CKD progression and to delay the need for RRT [1,2,3].

In most cases, the pathophysiology of CKD involves an imbalance in favor of maladaptive repair after an initial renal insult that could be from metabolic, hemodynamic, or immunological origin, among other causes. Regardless of the etiology of renal injury, when a number of nephrons is destroyed, remaining units become overloaded. Compensatory glomerular hyperfiltration and hypertrophy contribute to the progressive loss of more and more nephrons, leading to the progression of renal disease [4]. Systemic and intra-renal overactivity of the renin–angiotensin–aldosterone system (RAAS), with consequent sodium retention, vasoconstriction, local inflammation, and fibrosis, usually go hand in hand with the progression of CKD, thus contributing to the renal function loss [5,6].

For the last 2 decades, the conservative treatment of CKD was mainly based on the pharmacological suppression of RAAS, with both AII-converting enzyme (ACE) inhibitors (ACEIs) and AII receptor blockers (ARBs), such as Losartan, associated to the control of CKD-predisposing factors (hypertension, hyperglycemia, hypercholesterolemia) and to the establishment of a healthy life style, with physical activity and a healthy diet. Until the beginning of the development of the present study, RAAS blockade was still the gold-standard pharmacological approach for clinical CKD management, although not reversing kidney damage nor completely detaining CKD progression [7].

In the attempt of achieving the reversion of already established renal damage, a number of studies employing cell therapy with mesenchymal stem cells (MSCs) as a potential therapeutic factor to treat experimental CKD have come out in the last years [8,9,10]. In this context, we have recently demonstrated that the association of a single renal subcapsular injection of MSC derived from adipose tissue (ASC) to the oral treatment with Losartan promoted significant improvement in the renoprotection usually achieved with Losartan in monotherapy in a severe model of advanced CKD, induced by 5/6 renal ablation (Nx model) in Wistar rats. When associated to the RAAS blockade, cell therapy with 2 × 10^6^ ASC promoted the complete normalization of proteinuria and albuminuria, as well as the regression of glomerulosclerosis and the attenuation of renal inflammation and fibrosis, strongly suggesting cellular therapy with ASC to be a potential adjuvant to the current therapeutic approach used for conservative CKD management [11].

Differently from what was believed in the early 2000s, when the first studies investigating the therapeutic potential of MSC administration started, the current literature has been demonstrating that MSCs may not exert their beneficial effects through direct cell differentiation and tissue replacement, but through indirect paracrine effects. According to recent studies, under local stimulation of the injured tissue, MSCs would produce and secrete extracellular vesicles (EVs) containing growth factors, chemokines, and cytokines, among other still unknown bioactives, which would act as mediators of cell–cell communication in the inflammatory microenvironment, thus promoting the modulation of inflammation in the host tissue and reducing the stimulus to fibrogenesis. Due to its potential immunomodulatory effects, EVs derived from MSCs have become one of the most explored focuses in the study of therapy with cellular products applied to many chronic degenerative diseases [12,13]. In line with this theory, Ebrahim and collaborators recently demonstrated that intravenous application of EVs derived from bone marrow MSCs (BMSCs) limited glomerular structural damage and tubulointerstitial fibrosis in rats subjected to a model of streptozotocin-induced diabetic nephropathy [14].

Based on the promising results obtained by Maires and collaborators with the application of ASC in the renal subcapsular space of rats subjected to the Nx model, the general hypothesis of the present study is that the application of EVs derived from ASCs can promote the same renoprotective effect observed with the application of whole cells, thus confirming the protective effect of cell therapy derives from the paracrine factors released by these cells in the injured microenvironment.

## 2. Materials and Methods

### 2.1. Animal Model

All the experimental procedures included in the present study were approved by the Research Ethics Committee for the Use of Experimental Animals of the University of São Paulo Medical School (CEUA FMUSP No 1761/2022).

One-hundred and ten male albino Wistar rats, weighing 220–280 g, were purchased from the animal facility of the University of São Paulo Institute of Biomedical Sciences (Biotério de Produção de Ratos do ICB-USP). Rats were kept under controlled temperature (23 ± 1 °C), relative air humidity (5%), and artificial light/dark cycle (12/12 h), and had free access to conventional rodent chow and tap water for the duration of the experimental protocol. Animals were identified and had their body weight (BW, g) measured on alternate days throughout the experiment period. Systolic blood pressure (SBP, mmHg) was assessed by the tail cuff method, with an automated device (BP-2000 Blood Pressure Analysis System^TM^, Visitech Systems, Apex, NC, USA), at 3 different moments of the protocol: before CKD induction, 15 and 30 days after renal ablation; 24 h urine samples were also collected to evaluate the urinary flow (UV, mL), urinary protein excretion (UPE, mg/24 h), and urinary albumin excretion (UAE, mg/24 h) rates.

Ninety of the above-mentioned rats underwent surgical 5/6 renal ablation for CKD induction. Animals were subjected to isoflurane inhalation anesthesia (BioChimico, Rio de Janeiro, RJ, Brazil), underwent a ventral laparotomy, and had two of the three branches of the left renal artery ligated, resulting in the infarction of two-thirds of the organ. Total nephrectomy of the right kidney was performed and, after appropriate suture in two tissue planes, rats received SC injections of analgesic (5 mg/kg of Tramadol) and IM injection of antibiotic: 0.4 mL/kg Enrofloxacin 5% (Bayer, Leverkusen, NW, Germany). Additional 20 rats, used as controls, were submitted to the same inhalation anesthesia and ventral laparotomy, with no kidney manipulation (Sham). After surgery, rats were kept in heated cages until recovering from anesthesia and analgesic applications were repeated every 12 h for 2 further days.

### 2.2. Rat ASC Isolation and Characterization

Adipose-derived mesenchymal stromal cells (ASCs) were isolated from the perigonadal adipose tissue of healthy adult male albino Wistar rats, as previously described [11]. Briefly, during the CKD induction surgery, 3 sham animals had small samples of its perigonadal adipose tissue removed, minced, enzymatically digested, and centrifuged. The obtained cell pellet was resuspended in complete DMEM-Low medium (15% FBS), plated in culture flasks and kept in a humid oven at 37 °C, 5% CO_2_ (Thermo Fisher Scientific, Marietta, GA, USA), under daily observation and culture medium changes, twice a week. Cell passages for culture expansion were performed whenever the flasks reached 80% of confluence.

**Figure 1:**
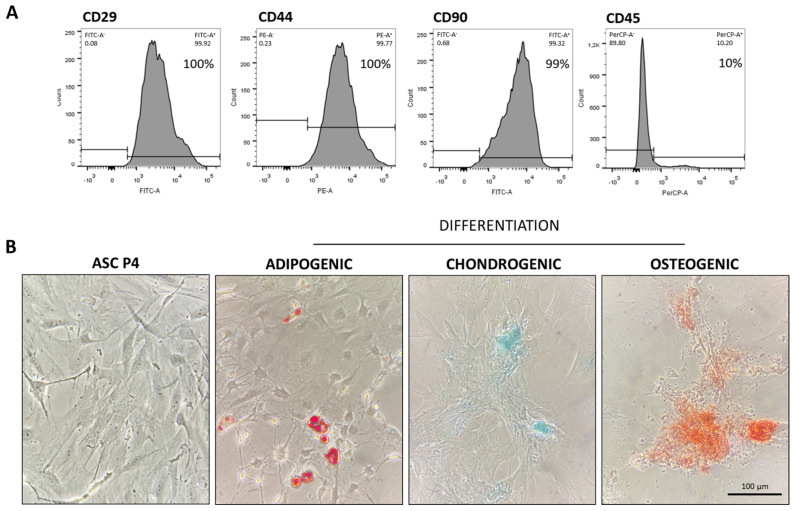
Characterization of ASCs at the 4th cell passage: (**A**) ASCs were immunophenotyped by flow cytometry, through which we observed that the cell population was 100% positive for CD29 and for CD44, and 99% positive for CD90, typical mSC biomarkers. Moreover, only 10% of these cells were positive for CD45, a surface protein expressed in hematopoietic cells and employed as a negative biomarker in mSC culture. (**B**) ASCs at P4 were also subjected to cell plasticity tests in order to assess the cell ability to differentiate when receiving appropriate culture media. As shown in the illustrative microphotographs, depending on cell media, ASCs can differentiate into adipogenic, chondrogenic, and osteogenic cell lineages, exhibiting lipid droplets stained in red, sulfated matrix proteoglycans stained in turquoise blue, or reddish calcium crystals, respectively.

ASCs in the 4th passage (P4 ASC) were immunophenotyped by flow cytometry BD FACSCanto II (Becton Dickinson, San Jose, CA, USA, USA). For this characterization, the presence of specific cellular surface markers CD29, CD44, and CD90 as well as the absence of the pan-leukocyte marker CD45 were verified using specific monoclonal antibodies [15]. Resulting histograms are shown in Figure 1A. Additionally, in the same cell passage, ASCs underwent cell plasticity tests to verify their ability to differentiate into osteogenic, chondrogenic, and adipogenic cell lines, using the commercially available kit STEMPRO^®^ Osteocyte/Condrocyte/Adipocyte Differentiation (Thermo Fisher Scientific, Marietta, GA, USA). These results were shown in Figure 1B.

### 2.3. Isolation and Characterization of EV Derived from ASC

EV inoculums were obtained from the supernatant of 2 culture flasks of 75 cm^2^ containing 1 × 10^6^ P4 ASC each (2 × 10^6^ P4 ASC in total). For this purpose, cells were kept under FBS deprivation for 24 h, when the conditioned culture medium was collected and subjected to a sequence of centrifugations at 4 °C in a Himae CP80NX ultracentrifuge, using a P50AT2 fixed-angle rotor (Hitachi Koki Co., Tokyo, Japan). A schematic representation of complete workflow for EV obtaining can be seen in Supplementary Figure S1. First of all, cell medium was centrifuged at 300 G for 10 min, followed by 3000 G for 20 min, to remove eventual remaining cells. The medium was then centrifuged at 100,000 G for 120 min, and a pellet containing the EV was obtained. This pellet was carefully washed with PBS and centrifuged again at 100,000 G for 30 min to purify the EV extract.

**Figure 2:**
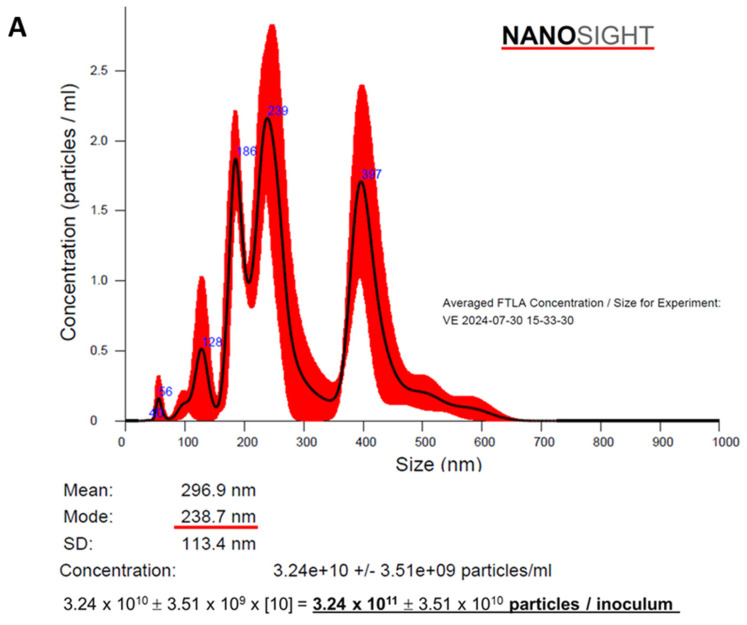

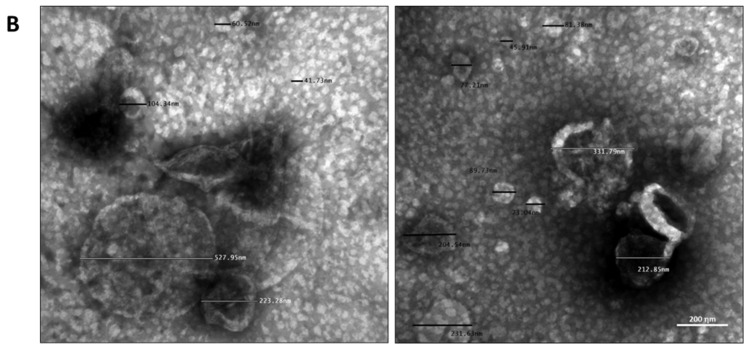
Characterization of EVs obtained from ASCs: (**A**) The concentration and size distribution of EVs were analyzed using a NANOSIGHT 3 Nanoparticle Tracking Analysis (NTA) device (NanoSight Ltd.). After the dilution factor correction, we found that each EV inoculum was composed of approximately 3 × 10^11^ particles, varying in size between 50 and 600 nm, with a predominant population of particles with an approximate diameter of 240 nm (mode)~270 nm (mean). (**B**) Obtained EVs were morphologically characterized by electron transmission microscopy, using Electron Transmission Microscope model JEM 1011-JEOL/MA/USA at 80 kV.

The concentration and size distribution of obtained particles was analyzed in the NANOSIGHT 3 Nanoparticle Tracking Analysis (NTA) device (NanoSight Ltd, Salisbury, Wiltshire, UK). To ensure accuracy, 3 reading repetitions of each sample were performed, under [1:100] dilution, and the obtained means and modes were shown in Figure 2A. The EV solution employed in the present study was composed by a pool of particles with sizes from 50 to 600 nm, with a predominant population of particles with an approximate diameter of 240 nm (mode)~270 nm (mean), indicating that it was mainly composed by microvesicles (100–1000 nm) with a small number of exosomes (30–100 nm) [12,13,16]. Moreover, the concentration of particles in the solution was verified and after dilution factor correction, each EV inoculum was demonstrated to be composed by approximately 3 × 10^11^ (mean) ± 3 × 10^10^ (SD) particles. In parallel, the total protein content of obtained EV pellets was determined by a colorimetric technique (Pierce™ BCA Protein Assay Kit #23227, Thermo-Fisher Scientific), after standard RIPA protein extraction protocol. Total protein dosage was performed in 5 different samples of EV inoculums and the average protein content was 143 (mean) ± 14 (SE) µg per inoculum (Supplementary Table S1). Finally, the EV extracts were morphologically characterized by electron transmission microscopy using Electron Transmission Microscope model JEM 1011 (JEOL, Peabody, MA, USA), at 80 kV. Digital microphotographs were acquired with Gatan software version 1.6, model 785 ES1000W (Gatan, Pleasanton, CA, USA) and are represented in Figure 2B.

### 2.4. Experimental Protocol

After 15 days of renal ablation, CKD animals were equally randomized, according to their results of SBP and UPE, into 5 experimental groups: basal CKD (N = 15), euthanized 15 days after CKD induction; CKD (N = 22), kept untreated until the 30th day after 5/6 renal ablation; CKD LOS (N = 22), which received 50 mg/kg/day of Losartan, diluted in drinking water, from the 15th to the 30th days after CKD induction; CKD EV (N = 15), which received a subcapsular injection of approximately 3 × 10^11^ EV, derived from 2 × 10^6^ ASCs, after 15 days of renal ablation, and were followed until the 30th day after CKD induction and CKD LOS + EV (N = 15), which received both the EV subcapsular injection and the oral treatment with Losartan (Supplementary Figure S2).

For EV delivery, recipient animals were submitted to a second open surgery after 15 days of CKD induction. Rats where anesthetized with isoflurane again, underwent ventral laparotomy and were submitted to the subcapsular injection of the EV inoculum [8]. Postoperative care was similar to the performed after 5/6 renal ablation. At the end of this study, 30 days after renal ablation, BW, SBP, UV, UPE, and UAE were reevaluated. Further, 24 h urine samples were collected to determine the creatinine clearance (CrCl) and the urine sodium (UNa, mEq/L) and potassium (UK, mEq/L) concentrations. The animals were once more anesthetized, submitted to a xiphopubic laparotomy, and euthanized by exsanguination. Blood samples were collected to assess serum creatinine (SCr, mg/dL) and urea (SUr, mg/dL) concentrations, using commercially available kits (Creatinina #35 Kit and Urea CE #27 Kit, Labtest, Lagoa Santa, MG, Brazil), as well as for serum sodium (SNa, mEq/L) and potassium (SK, mEq/L) determination. Estimated creatinine clearance (CrCl, mg/min) was obtained by [(Ucr × UV)/Scr]/1440. Further corrections for rat body surface area (RBSA ≅ 357 cm^2^) were obtained by dividing result by 0.0357 (CrCl, mg/min/RBSA). The fractional excretions of sodium (FENa, %) and potassium (FEK, %) were obtained by (UNa/SNa)/(UCreat/SCreat) × 100 and (UK/SK)/(UCreat/SCreat) × 100, respectively.

The left kidney was removed and weighted. Left kidney weight (LKW, g) was employed to assess the renal hypertrophy index through the following calculations: (LKW/BW) × 10^3^. Half of it was chemically fixed for histological and immunohistochemical analysis, and the remaining half was quickly frozen in liquid nitrogen and kept at −80 °C for complementary gene expression assessment.

### 2.5. Renal Histological and Immunohistochemical Analysis

Renal samples were prefixed in Du Boscq-Brasil, followed by fixation with buffered paraformaldehyde. Fixed fragments were sequentially dehydrated, diaphanized, and included in paraffin blocks from which histological sections of 4 μm were obtained. For histological and immunohistochemical analyses, tissue slides were deparaffinized and rehydrated. Periodic Acid–Schiff (PAS) histological staining was performed to analyze the glomerular architecture of the kidney samples from the animals of the different experimental groups. The percentage of glomerulosclerosis (GS%) was assessed by the blinded analysis of 50 glomeruli of each animal, under a final magnification of 400×. Masson’s trichrome histological staining, in turn, was employed to evaluate the presence of renal cortical interstitial fibrosis in the kidney samples of animals from the studied group. The percentage of interstitial fibrosis was determined in 30 consecutive microscopic fields, under a final magnification of 200× [17].

Immunohistochemistry was employed to detect myofibroblasts, by the presence of α-smooth muscle actin (α-SMA) deposition in the renal cortical interstitial area, as an additional fibrosis biomarker. This technique was also used to assess renal infiltrating macrophages (CD68) and proliferating cells (PCNA) to investigate tissue inflammation and to identify and quantify the constitutive glomerular protein Zonula Occludens-1 (ZO-1), related to the integrity of the glomerular barrier membrane. After dewaxing and antigen retrieval, renal slides underwent immunophosphatase for α-SMA and CD68 detection, or immunoperoxidase for PCNA, using the respective primary antibodies: Monoclonal mouse anti α-SMA (#A2547, Sigma, San Luis, MO, USA); anti-CD68 (#MCA341R, AbD Serotec-Bio Rad, Hercules, CA, USA); anti-PCNA (#M0879 Dako-Agilent, Santa Clara, CA, USA); and anti-ZO-1 (#617300, Zymed-Thermo Fisher Scientific, Marietta, GA, USA). Both interstitial infiltration by macrophages and the number of proliferating cells were evaluated by counting the positive cells for CD68 and PCNA under a final 400× magnification in at least 30 microscopic fields per animal. The percentage of renal interstitial α-SMA was achieved using the same point-counting technique employed to quantify Masson’s trichrome positivity. The percentage of glomerular area occupied by ZO-1 was evaluated in at least 25 glomeruli, under 400× magnification.

### 2.6. Local Renal Gene Expression

In order to evaluate and compare the local gene expression of interleukins *Il-1β*, *Il-2*, *Il-4*, *Il-6*, and *Il-10* in the renal cortex of animals from the different experimental groups, RT-qPCR analysis was performed. As previously described, renal tissue samples obtained from 10 animals from each experimental group were submitted to Trizol-based RNA extraction (#15596018, Ambion-Thermo Fisher Scientific, Marietta, GA, USA). Obtained RNA extracts were subjected to concentration and purity analyses through spectrophotometry using the Thermo Scientific™ NanoDrop™ 2000/2000c device (ThermoFisher Scientific). Remaining gDNA elimination, with a Turbo DNAse, RNAse free™ kit (#AM1907, Invitrogen-Thermo Fisher Scientific, Marietta, GA, USA), was carried out, followed by an analysis of RNA integrity through agarose gel electrophoresis, in which ribosomal 18S and 28S RNA bands could be seen when treated with SYBR Safe nucleic acid gel stain (Thermo Fisher Scientific, Marietta, GA, USA). Reverse-transcriptase reaction was performed in samples of 1 µg of total RNA, using the Oligo(dT)15 Primer with the M-MLV RT enzyme (#M1705, Promega Corporation, Madison, WI, USA). After cDNA synthesis, qPCR reactions were conducted in triplicates of each sample with specific pairs of primers (Supplementary Table S2) using the Syber GreenER qPCR SuperMix Universal (Invitrogen #11762) in the StepOne Plus platform (Applied Biosystetems-Thermo Fisher Scientific, Marietta, GA, USA). Actin-β (Bact) was used as the constitutive normalizing gene. Non-RT RNA samples were used as negative controls. Target genes had their expressions quantified by the comparative ∆∆Ct method [11].

### 2.7. Statistical Analysis

Results are presented as mean ± SE. All statistical analysis was performed using GraphPad Prism^®^ software version 7.0. One-way analysis of variance (ANOVA) followed by the appropriate Tukey or Dunnett post-test was performed to compare the groups, and *p* values under 0.05 were considered significant [18].

## 3. Results

### 3.1. LOS + EV Reverts Polyuria, Creatinine Retention, and Hypertension

As can be seen in Table 1, all rats underwent the 5/6 renal ablation model exhibited lower body weight, intense polyuria, serum retention of creatinine and urea, and compensatory hypertrophy of remnant left kidney mass compared to the sham group at both 15 and 30 days after CKD induction. The association of a single inoculation of EV to the oral treatment with LOS significantly reduced polyuria and serum creatinine concentration in these animals.

**Figure 3:**
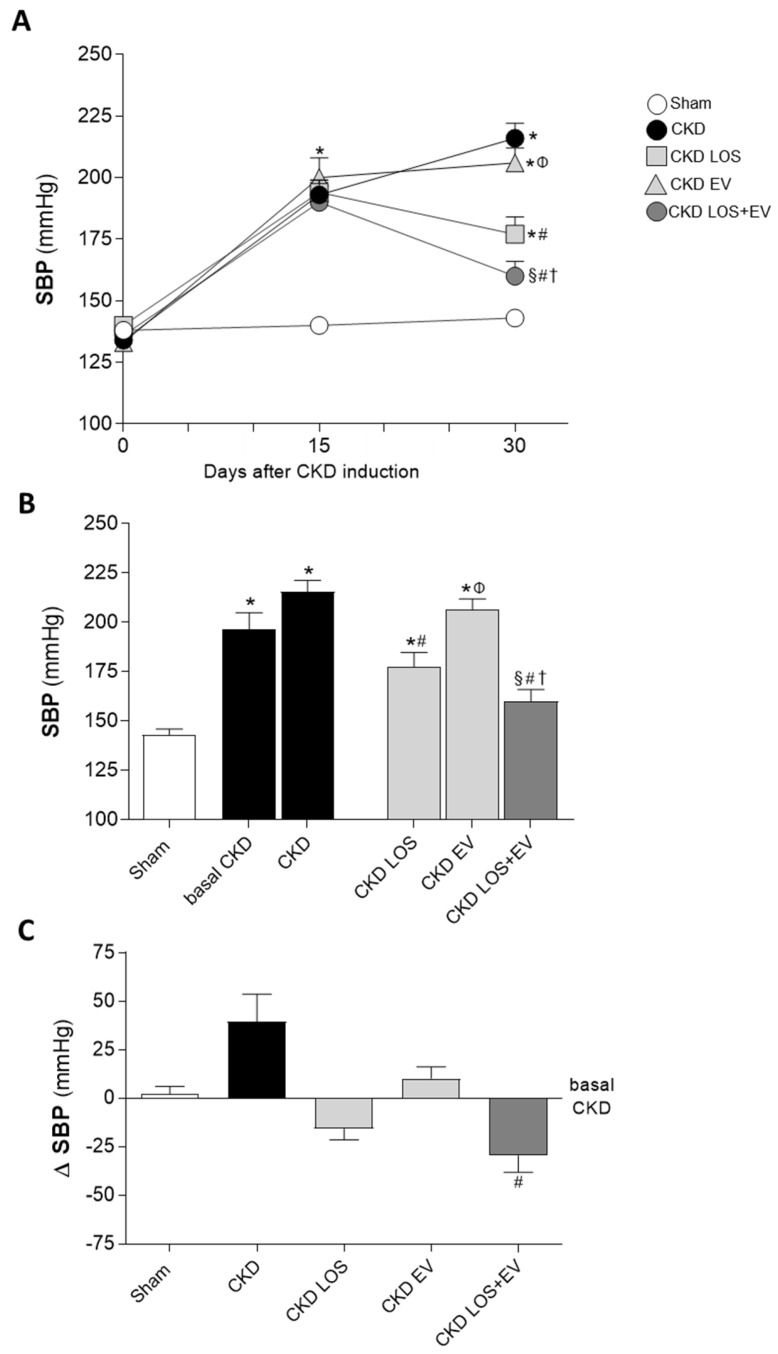
Systolic blood pressure (SBP, mmHg): (**A**) Line graphs showing the time course of SBP in the different experimental groups, throughout the protocol. (**B**) Bar graphs of SBP after 30 days of CKD induction. (**C**) Delta bar graphs, obtained by subtracting the values observed at 30 days from those obtained at 15 days, before the beginning of treatments. Statistical differences are * *p* < 0.05 vs. sham, § *p* < 0.05 vs. basal CKD, # *p* < 0.05 vs. CKD, Φ *p* < 0.05 vs. CKD LOS, and † *p* < 0.05 vs. CKD EV.

Illustrative line graphs of SBP evaluation throughout this study (before CKD induction and 15 and 30 days after renal ablation) can be seen in Figure 3A, while bar graphs of SBP at the end of protocol are shown in Figure 3B. Further analysis of the ΔSBP, obtained from the subtraction (final SBP, at 30 d of CKD)—(basal SBP, at 15 d of CKD induction), was also performed and is presented in Figure 3C. Corroborating the literature, Wistar rats submitted to the 5/6 renal ablation model already presented significant hypertension 15 days after CKD induction when compared to time-paired sham animals (194 ± 3 vs. 140 ± 3 mmHg, *p* < 0.05). In both untreated and EV-treated CKD rats, hypertension worsened with time, reaching the exuberant values of 216 ± 6 and 206 ± 6 mmHg 30 days after renal ablation, respectively. As expected, the oral treatment with Losartan significantly reduced the hypertension in CKD LOS group compared to untreated animals (177 ± 7 vs. 216 ± 6 mmHg); however, this parameter remained statistically higher than that observed in sham 30 days after surgery (143 ± 3 mmHg, *p* < 0.05). Notably, the association of a single subcapsular application of EV derived from ASC to the Losartan treatment promoted an additional reduction in SBP, leading to the statistical reversion of hypertension in the CKD LOS + EV group (160 ± 6 vs. 143 ± 3 mmHg in sham rats, *p* > 0.05).

### 3.2. EV Combined to Losartan Promotes Additional Reduction of Proteinuria and Albuminuria and Prevented Glomerulosclerosis in Rats Undergoing 5/6 Renal Ablation

**Figure 4:**
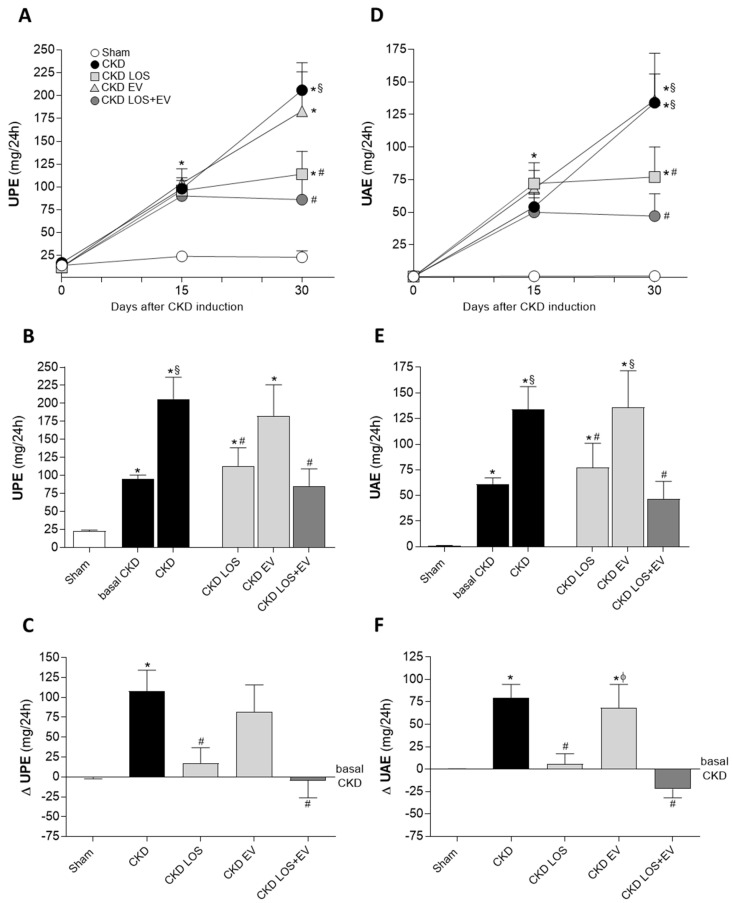
Urinary protein (UPE, mg/24 h) and albumin (UAE, mg/24 h) excretion of the animals of each experimental group: Line graphs show the time course of UPE (**A**) and UAE (**D**) in the different groups, throughout the protocol. Bar graphs show UPE (**B**) and UAE (**E**) after 30 days of CKD induction. Finally, delta bar graphs for UPE (**C**) and UAE (**F**) were obtained by subtracting the values observed at 30 days from those obtained at 15 days, before the beginning of treatments. Statistical differences are * *p* < 0.05 vs. sham, § *p* < 0.05 vs. basal CKD, # *p* < 0.05 vs. CKD, Φ *p* < 0.05 vs. CKD LOS.

As demonstrated in Figure 4A,D, all rats submitted to the 5/6 renal ablation model developed significantly proteinuria and albuminuria after 15 days of CKD induction compared to the sham group (UPE: 96 ± 6 vs. 24 ± 2 and UAE: 62 ± 6 vs. 1 ± 1 mg/24 h, *p* < 0.05). Moreover, all CKD groups exhibited very similar UPE and UAE mean values at this point, before starting the different treatments. In untreated CKD rats, both proteinuria and albuminuria raised vertiginously over time, reaching 206 ± 30 and 134 ± 22 mg/24 h, respectively, after 30 days of renal ablation. As expected, the administration of LOS promoted a deceleration in the progression of proteinuria and albuminuria in CKD LOS group in which the final mean values of UPE and UAE were 114 ± 25 and 77 ± 23 mg/24 h, respectively. However, as shown in Figure 4B,E, proteinuria and albuminuria exhibited by CKD LOS animals were still significantly higher than those observed in sham rats after 30 days of follow-up (23 ± 2 and 1 ± 1 mg/24 h). Curiously, although promoting no renoprotection when administered alone, if associated to LOS oral treatment, a single EV subcapsular injection enhanced the beneficial effects of LOS and led to a further reduction in UPE (86 ± 24) and UAE (47 ± 17) mean values in the LOS EV group, which were not statistically different from the means exhibited by sham rats. Delta graphs were built up in order to better illustrate this additional effect of EVs in UPE and UAE reduction. According to Figure 4C,F, the LOS + EV combination was the only effective approach to reverse proteinuria and albuminuria to values below those observed before the beginning of treatments. Curiously, is spite of this reduction in UPE and UAE rates, none of the tested treatments could revert the decrease in the percentage of ZO-1 in the glomeruli of CKD animals (Supplementary Figure S3).

**Figure 5:**
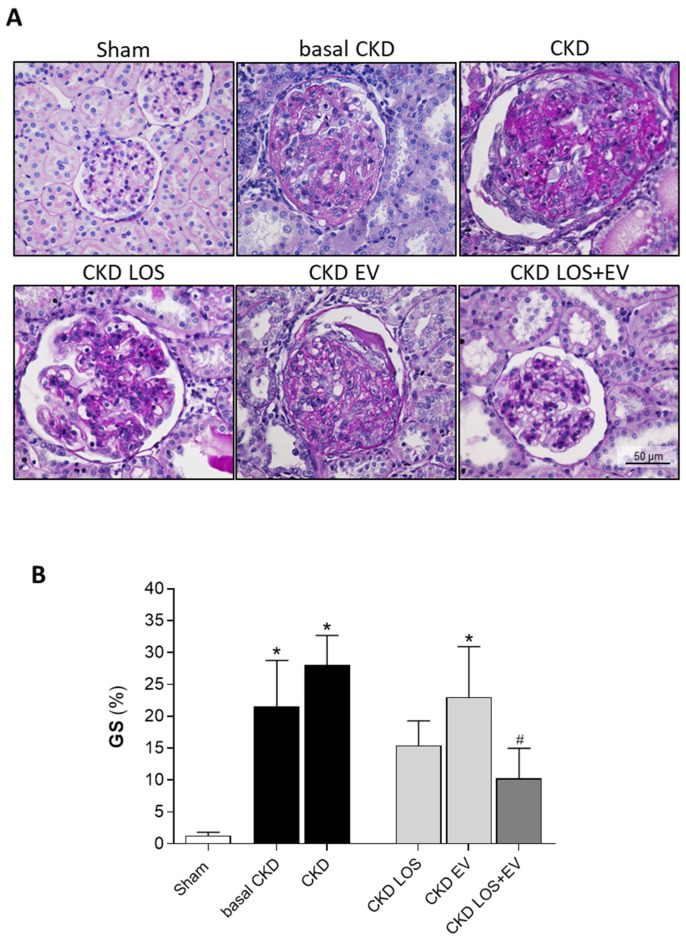
Glomerular damage: (**A**) Illustrative microphotographs of PAS-stained sections of each experimental group under final 400× magnification. (**B**) Bar graphs showing the percentage of glomerulosclerosis (GS%) in the different groups by the end of the protocol. Statistical differences are * *p* < 0.05 vs. Sham, # *p* < 0.05 vs. CKD. EV associated with LOS promotes further attenuation of renal fibrosis.

Additionally, glomerular structural damage was evaluated in PAS-stained renal sections of animals from each experimental group. Representative microphotographs of studied glomeruli, under a final 400× magnification, can be seen in Figure 5A, while the quantification of the percentage of glomerulosclerosis (GS%) in each group is presented as a bar graph in Figure 5B. As clearly demonstrated, rats that underwent the 5/6 renal ablation model exhibited severe glomerulosclerosis as early as 15 days after CKD induction when compared to sham rats (22 ± 7 vs. 1 ± 1%, *p* < 0.05). GS% worsened over time in untreated CKD animals (28 ± 18%) and remained stable in CKD rats that received only the experimental EV application (23 ± 5%). As expected, LOS monotherapy promoted a numerical reduction in the percentage of glomerulosclerosis compared to basal CKD (15 ± 7%); however, the reduction in GS% was only statistically significant in the group of animals treated with both LOS and EV in association (10 ± 1% vs. 28 ± 18% in untreated CKD rats, *p* < 0.05).

### 3.3. EV Associated with LOS Promotes Further Attenuation of Renal Fibrosis 

**Figure 6:**
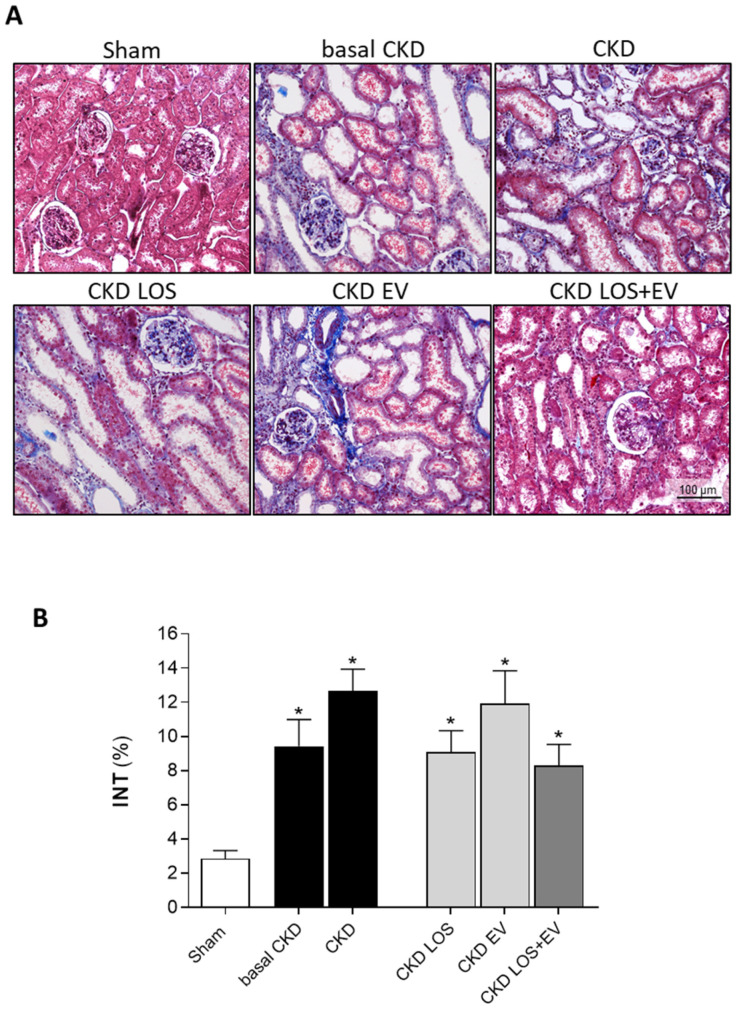
Interstitial fibrosis: (**A**) Illustrative microphotographs of Masson’s trichrome-stained sections of each experimental group under final 200× magnification. (**B**) Bar graphs showing the percentage of interstitial fibrosis (INT%) in the different groups by the end of the protocol. Statistical differences are * *p* < 0.05 vs. sham.

**Figure 7:**
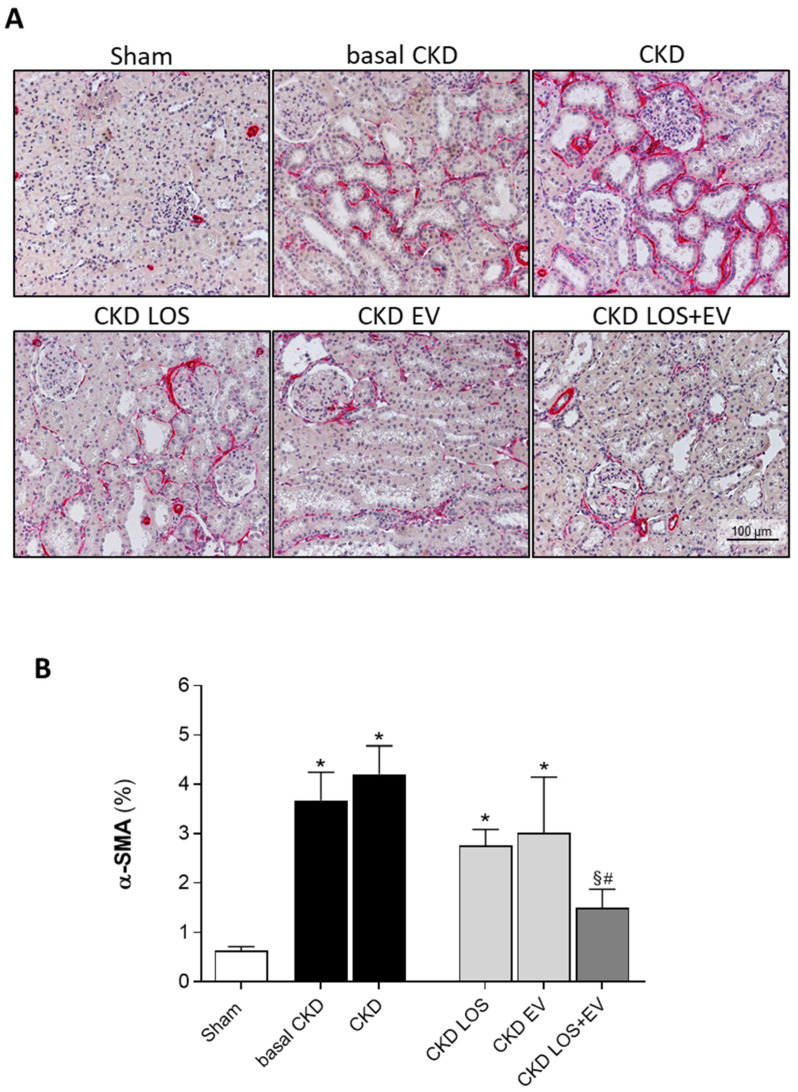
Interstitial myofibroblasts: (**A**) Illustrative microphotographs of immunohistochemistry for α-SMA, used to detect myofibroblasts, in renal cortical sections of each experimental group, under final 200× magnification. (**B**) Bar graphs showing the percentage of interstitial area occupied by α-SMA in the different groups by the end of the protocol. Statistical differences are * *p* < 0.05 vs. sham, § *p* < 0.05 vs. basal CKD, # *p* < 0.05 vs. CKD.

Interstitial fibrosis (INT%) was evaluated in renal samples of all animals of each experimental group. Cortical sections were stained with Masson’s trichrome technique, and the percentage of fibrosis was determined by the fraction of renal interstitial area positively stained in blue (collagen), under a final 200× magnification. Illustrative microphotographs of stained sections can be seen in Figure 6A, while the bar graphs representing the quantification of this parameter are shown in Figure 6B. In accordance with the literature, we observed that our animals subjected to the remnant model exhibited significant renal fibrosis as soon as 15 days after CKD induction (9 ± 2 vs. 3 ± 1% in sham rats, *p* < 0.05). The percentage of fibrosis worsened progressively in the untreated group over time, reaching 14 ± 2% at 30 days after 5/6 renal ablation. Losartan monotherapy prevented the progression of INT% (9 ± 1%), and the association of EV inoculation to this treatment promoted only a slight numerical decrease in the percentage of fibrosis compared to the result obtained with LOS alone. More exciting results were observed when analyzing the presence of renal interstitial myofibroblasts through the quantification of the percentage of cortical interstitial area occupied by α-SMA in the renal parenchyma of studied animals. Illustrative microphotographs of immunohistochemistry for α-SMA can be seen in Figure 7A. Once more, CKD rats presented significant interstitial accumulation of myofibroblasts at 15 days after renal ablation (3.7 ± 0.6 vs. 0.6 ± 0.1% in sham rats, *p* < 0.05), which raised only numerically after 30 days of CKD induction in the untreated group (4.2 ± 0.6%). While the monotherapies with both Losartan or EV only partially detained the renal myofibroblast infiltration (2.8 ± 0.3 and 3.0 ± 1.1, respectively), the association of LOS + EV reversed the accumulation of interstitial α-SMA to values lower than those observed in basal CKD group and statistically similar to those observed in sham animals (1.5 ± 0.4 vs. 0.6 ± 0.1% in sham rats, ns). The bar graphs illustrating these results are presented in Figure 7B.

### 3.4. LOS + EV Reduces Inflammation and Promotes the Upregulation of Il-4

**Figure 8:**
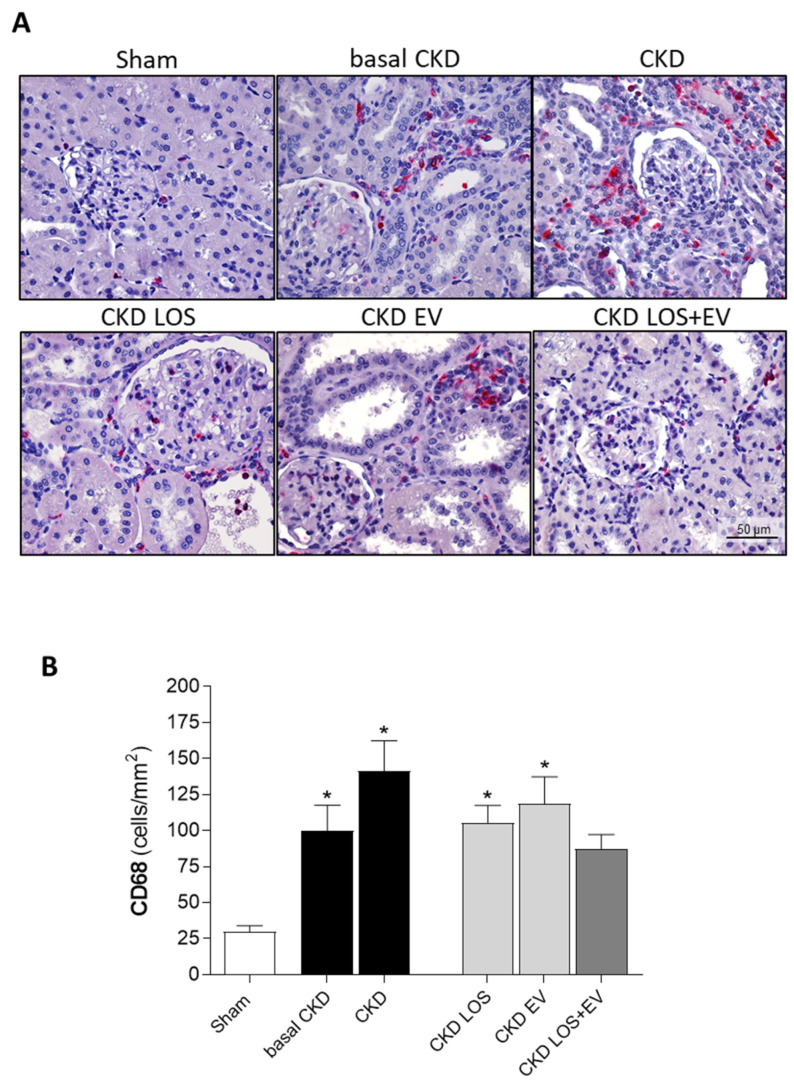
Renal interstitial macrophage infiltration: (**A**) Illustrative microphotographs of immunohistochemistry for CD68 in renal cortical sections of each experimental group under final 400× magnification. (**B**) Bar graphs showing the mean number of interstitial CD68+ cells/mm^2^ of renal cortical area of animals from the different groups by the end of the protocol. Statistical differences are * *p* < 0.05 vs. sham.

**Figure 9:**
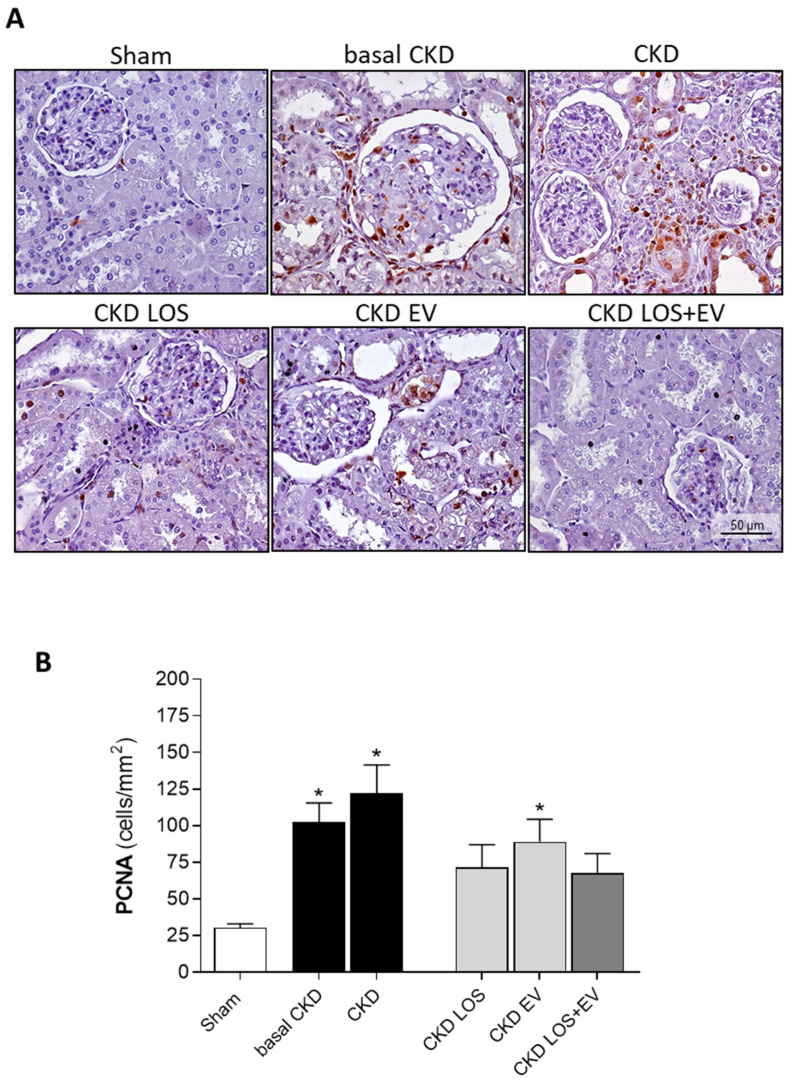
Local renal inflammation—Interstitial PCNA+ proliferating cells: (**A**) Illustrative microphotographs of immunohistochemistry for PCNA in renal cortical sections of each experimental group under final 400× magnification. (**B**) Bar graphs showing the mean number of interstitial proliferating cells/mm^2^ of renal cortical area of animals from the different groups by the end of the protocol. Statistical differences are * *p* < 0.05 vs. Sham.

**Figure 10:**
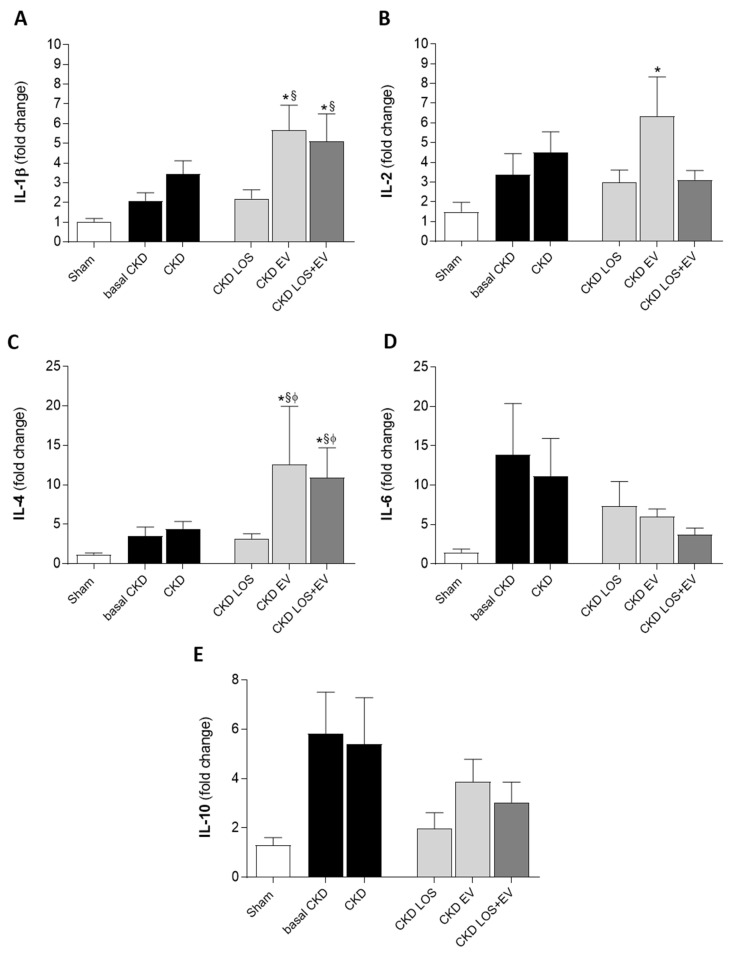
Bar graphs illustrating the quantification of renal gene expression (RT-qPCR) of interleukins (**A**) *Il-1β*, (**B**) *Il-2*, (**C**) *Il-4*, (**D**) *Il-6*, and (**E**) *Il-10*. Statistical differences are * *p* < 0.05 vs. sham, § *p* < 0.05 vs. basal CKD, Φ *p* < 0.05 vs. CKD LOS.

In the present study, we assessed the local renal interstitial inflammation in the animals of each experimental group by immunohistochemistry and RT-qPCR. Through the first technique, the number of infiltrating CD68+ macrophages as well as the amount of the proliferating PCNA+ interstitial cells were evaluated. Illustrative microphotographs of the immunohistochemistry for CD68+ macrophages and for PCNA+ interstitial cells can be found in Figure 8A and Figure 9A, respectively. As can be seen in the bar graphs of Figure 9B and Figure 10B, both macrophage infiltration and interstitial proliferation were already statistically increased in rats submitted to the 5/6 renal ablation model after 15 days of CKD induction (100 ± 18 and 102 ± 13 vs. 30 ± 4 and 30 ± 3 cells/mm^2^ in sham rats, respectively. *p* < 0.05). After 30 days of 5/6 ablation, the local inflammation progressed in the renal cortex of untreated rats. Macrophage infiltration reached 141 ± 21 cells/mm^2^, while the number of PCNA+ interstitial cells was 122 ± 20 cells/mm^2^ in this group. The therapeutic association of Losartan to a single local renal inoculation of EVs derived from ASCs promoted superior anti-inflammatory activity compared to the oral monotherapy with Losartan. In the LOS + EV group, the interstitial macrophage infiltration was reduced to 87 ± 10, while the number of observed proliferating cells was only 68 ± 13 (Figure 8B and Figure 9B).

According to our RT-qPCR results, presented in Figure 10, local renal overexpression of genes coding for the pro-inflammatory interleukins *Il-1β*, *Il-2*, *Il-6*, and *Il-10* was noticed in untreated CKD rats as soon as 15 days after renal ablation, raising with time in almost all the observed targets. Unexpectedly, LOS + EV association promoted a further increase in *Il-1β* expression while reducing the expression of all other pro-inflammatory genes: *Il-2*, *Il-6*, and *Il-10*. Additionally, renal EV inoculation alone or in addition to Losartan treatment led to an increased expression of the anti-inflammatory interleukin *Il-4* in groups EV and LOS + EV.

## 4. Discussion

As described in the literature, the experimental CKD model based on the surgical 5/6 nephrectomy (Nx) leads to the development of an important nephropathy that bears many similarities to human disease. Mimicking what is observed in clinical practice with patients in advanced stages of CKD, Nx rats presented a significant delay in weight gain throughout the observation period, regardless whether they received any therapeutic treatment. Therefore, at the end of this study, CKD animals had an average of 60 g less body weight when compared to healthy animals of the same age, probably reflecting a state of sarcopenia and metabolic disorder related to the loss of protein in the urine, caused by the severity of renal dysfunction [8,11,19,20].

Another feature shared by animals submitted to the 5/6 model and most patients with advanced-stage CKD is the development of sustained and resistant hypertension, which is known to be responsible for the increased risk of cardiovascular events in patients under conservative treatment or receiving RRT [21]. As in human nephropathy, our CKD animals exhibited significant hypertension as soon as 15 days after renal ablation, which worsened throughout this study in untreated animals, clearly demonstrating the temporal evolution of renal function impairment. As expected, Losartan monotherapy partially reduced SBP levels in CKD animals, but it was not sufficient to normalize this parameter. Although receiving the gold-standard treatment, CKD LOS animals remained hypertensive when compared to the sham group, thus reflecting the difficulty in managing the hypertension in severe cases of CKD [19,21]. Surprisingly, when associating a single renal application of EVs derived from ASCs to Losartan therapy, an additional reduction in SBP levels was observed. Although animals in the CKD LOS + EV group were not completely normotensive from a numerical point of view, they presented mean SBP values that did not differ statistically from those observed in the sham animals, pointing to a specific synergistic effect of LOS + EV association. Studies reporting the application of EV as therapeutic options in hypertension-associated kidney diseases are still limited. However, our findings are in line with the results obtained by Lindoso and collaborators. The authors demonstrated that multiple administration of EVs from ASCs exerted renoprotective effects and prevented the development of hypertension in an experimental model of deoxycorticosterone acetate (DOCA)-salt hypertensive renal damage [22].

Since the main contents of EVs are small noncoding RNAs (especially miRNAs), the latest literature reviews have been suggesting that a possible mechanism of action of EVs in preventing or detaining the progression of hypertension could lay on the negative regulation of host genes involved in SBP raising by these miRNAs delivered by EVs directly in the host tissue [13]. miRNA can control the expression of a specific gene by complementary binding with its mRNA in the cell cytoplasm, precluding the migration of the mRNA to the ribosome and the translation of it into a protein [23]. Due to its high potential as a tool for gene therapy in the near future, a number of miRNAs found in EVs produced by different cell types are currently being sequenced and characterized according to their function. miRNA-663, for instance, is known to regulate renin gene expression, thus potentially exerting antihypertensive effects [24]. Although further investigation must be conducted in order to identify and characterize the miRNA content of ASC-derived EVs employed as an adjuvant treatment to Losartan therapy in the present study, the presence of antihypertensive miRNA into the applied EVs could be a plausible explanation for the results we found.

Similar to the observed with the hypertension, the association of EVs to the treatment with LOS also exerted significant renoprotection regarding glomerular damage and renal inflammation/fibrosis. The animals subjected to the 5/6 experimental CKD model rapidly evolved with the loss of selectivity of the glomerular filtration barrier, evidenced by the significant loss of protein (specially albumin) in urine, detected after 15 days of CKD induction. When kept untreated, these animals showed a clear progression of this parameter, with an approximate 100% increase in both proteinuria and albuminuria between the 15th and 30th day, corroborating the literature [19,25]. In the CKD group receiving only Losartan, the proteinuria mean increased 20% and the albuminuria increased by less than 10% throughout the study period, between 15 and 30 days of follow-up, a significantly lower evolution than that presented by animals maintained without any treatment. More exuberant effects were seen with the association of EV to Losartan therapy. CKD LOS + EV animals presented a decrease of 5% in the proteinuria between the 15th and 30th day after CKD induction and a decrease of 22% in the albuminuria in the same period. Our results demonstrated, for the first time, that the subcapsular application of EVs derived from ASCs exerted additional renoprotection in reducing UPE and UAE in the 5/6 remnant model, thus suggesting this cell product could be considered as an interesting additive strategy to the currently treatment with Losartan in detaining proteinuria and albuminuria in advanced CKD. More than this, our findings confirm the idea that the antiproteinuric effects achieved by cell therapy with ASC and demonstrated in previous studies are, in fact, due to the paracrine release factors contained in the EVs secreted by ASCs in the renal host microenvironment, and not to the presence of the cells themselves in the renal parenchyma [10,11,26,27,28]. It is important to emphasize that these results are of extreme clinical relevance, since both proteinuria and albuminuria are essential clinical and laboratory factors in the evaluation of CKD progression in human patients. Albuminuria is closely associated with the progression of CKD in adults: while the worsening of this parameter correlates positively with a higher degree of comorbidities and a higher risk of mortality, maintaining low levels of albuminuria in patients with CKD leads to a reduced risk of mortality in this population [29,30,31].

Once again, mimicking the findings in human nephropathy, animals submitted to the CKD model induced by 5/6 renal ablation surgery presented serum retention of urea and creatinine due to progressive loss of renal function and consequent reduction in the capacity to clear uremic toxins [11,25]. In the present study, we did not observe a reduction in the serum retention of these toxins in animals treated only with the BRAT-1 Losartan, unlike what usually occurs in clinical practice, in which this drug is at least partially effective. It is extremely important to emphasize that, unlike humans, rodents are animals capable of actively secreting creatinine through the renal tubules, which means that its elimination through urine does not truly reflect the preservation of the glomerular filtration rate, but rather the combination of this and the anatomical and functional integrity of the tubulointerstitial compartment [32,33,34]. Surprisingly, the association of a single administration of EV to LOS treatment promoted a significant reduction in creatinine retention, thus confirming the synergistic renoprotective effect of this combination.

Both the loss of macromolecules through the glomerular barrier and the serum retention of uremic toxins, characteristic features of CKD, are related to histological changes and loss of tissue integrity of the renal parenchyma, which include the development of glomerular sclerosis and tubulointerstitial inflammation and fibrosis [6]. When analyzing the renal histology of animals submitted to the Nx model, it was possible to observe that there was a significant loss of typical renal architecture, as soon as 15 days after CKD induction, when the Nx animals already presented a high percentage of sclerotic glomeruli, with visible reduction of the glomerular filtration surface and areas of adhesions of the glomerular tuft to Bowman’s capsule (synechiae). These findings became even more pronounced after 30 days of CKD induction and the differences in relation to the sham group became even more evident, therefore signaling the progression of the severity of the disease during the study period.

Reduction in the glomerular filtration surface, impairment of the selectivity of the filtration barrier, and the development of glomerulosclerosis are quite common findings in a series of nephropathies. Among the factors that trigger it, we can mention the increase in hydrostatic pressure in the glomerular capillaries and the increase in the filtration rate in the remaining nephrons, phenomena that promote stretching of the glomerular capillary, with consequent activation of innate immunity pathways, in response to mechanical stress, culminating in the development of a local inflammatory reaction. Once the inflammatory microenvironment is established, leukocyte infiltration begins to occur in the glomeruli, as well as an increase in the proliferation rate of mesangial cells and the exacerbated production of mesangial extracellular matrix, which is deposited between the capillary loops, leading to glomerulosclerosis [6,35].

As expected, Losartan in monotherapy was partially effective in containing the development of glomerulosclerosis in the remnant model, but the association of this drug with a single inoculation of ASC-derived EVs reduced the percentage of glomerulosclerosis in group LOS + EV to values lower than those observed before the beginning of treatments and statistically similar to those observed in the control animals of the sham group. It is important to emphasize that, from a statistical point of view, the animals submitted to monotherapy with Losartan presented glomerular sclerosis values that did not differ from those observed in the animals kept without treatment, suggesting that the subcapsular application of EVs played an important local anti-inflammatory and antifibrotic role when associated with pharmacological RAAS blockade. These data are in line with our previous studies employing whole ASCs as a therapeutic tool and with those from Yang and collaborators, using an experimental model of focal segmental glomerulosclerosis (FSGS) in rats. According to the authors, intravenous infusion of bone marrow-derived MSCs reduced 24 h proteinuria and promoted the preservation of renal function in animals subjected to FSGS, in addition to promoting a decrease in the ratio between metallopeptidase Inhibitor 1 (TIMP1) and matrix metalloproteinase-9 (MMP9). The reduced TIMP-1/MMP9 ratio in the renal tissue of these rats may have strongly contributed to the reduction in extracellular matrix (ECM) deposition and, consequently, to detain the progression of FSGS [36].

As mentioned previously, as CKD progresses, the tubulointerstitial compartment of the renal cortex also becomes a target for the establishment of a chronic inflammatory process, which will have fibrosis as a likely outcome. Changes in this compartment resulting from the progression of nephropathy range from dilatation of the tubular lumen and increased rates of proliferation and apoptosis of tubular epithelial cells, with consequent impairment of ionic management along the tubules, to interstitial leukocyte infiltration, as well as activation and proliferation of resident fibroblasts and their differentiation into myofibroblasts, leading to increased secretion and accumulation of the ECM in this compartment, culminating in the appearance of fibrosis [5,31].

Tissue fibrosis is, therefore, a pathophysiological process resulting from chronic inflammation maintained for a prolonged period and can be characterized by the accumulation of ECM proteins, especially collagen, in the interstitial area of tissues, leading to loss of function of the affected organ. In the present study, after 15 days of CKD induction, the animals submitted to the experimental model already presented pronounced interstitial fibrosis, as well as the presence of tissue myofibroblasts, evidenced by the interstitial accumulation of α-SMA in the renal parenchyma. In addition, intense and significant macrophage infiltration and a significant increase in interstitial cell proliferation could also be seen in 5/6 animals at this observation point. Furthermore, local renal expression of pro-inflammatory interleukins IL1β, IL2, IL6, and IL10 could already be detected by RT-qPCR at only 15 days of CKD progression. All of these parameters progressed significantly between the 15th and 30th day points in animals maintained without treatment when compared to sham animals, indicating the worsening of tissue inflammation and the development of renal fibrosis. These findings corroborate data previously published by other authors in this same CKD model [5,37]. It is known that, once established, renal fibrosis is difficult to treat, since the dissolution of collagen accumulation in the interstitial space depends on an intricate mechanism of synthesis and secretion of metalloproteinases and other enzymes involved in tissue remodeling, and that this natural recovery only occurs upon cessation of local pro-fibrotic stimuli. In this context, Losartan in monotherapy could promote an only subtle numerical reduction in renal interstitial fibrosis, myofibroblast accumulation, and macrophage infiltration in Nx rats, while the association of a single subcapsular application of EVs to Losartan treatment lead to a significant reduction in renal myofibroblasts and infiltrating macrophages, accompanied by the downregulation of pro-inflammatory interleukins IL6 and IL10 and upregulation of anti-inflammatory interleukin IL4, mimicking the findings of our previous studies using whole ASCs. These results demonstrate the effectiveness of the association of cell therapy with pharmacological treatment and consolidating the idea that both ASCs and what derives from these cells could be capable of promoting equivalent anti-inflammatory and antifibrotic effects [10,11,19,26,27,28,38].

In summary, when associated to Losartan treatment, a single subcapsular inoculation of EVs derived from ASCs promoted significant improvement in many parameters related to the progression of CKD in the 5/6 renal ablation model, thus promoting additional renoprotection when compared to Losartan given in monotherapy. This finding suggests that the administration of EVs as an adjuvant to pharmacological treatment of severe CKD may represent an interesting alternative to enhance clinical results, slowing down CKD progression and delaying the need for RRT.

## 5. Conclusions

In conclusion, our results suggests that the therapeutical application of a single dose of EVs derived from ASC in the renal sub-capsular area, associated to LOS treatment can promote additional renoprotective effects in an experimental model of severe CKD, when compared to the use of LOS alone. 

## Figures and Tables

**Table 1:** Final parameters analyzed at the end of this study: body weight (BW; g); urinary volume (UV; mL); serum creatinine concentration (SCr; mg/dL); urinary creatinine concentration (Ucr; mg/dL); creatinine clearance, corrected by the rat body surface area (CrCl; mg/min/RBSA); serum urea concentration (SUr; mg/dL); serum sodium concentration (SNa; mEq/L); urinary sodium concentration (UNa; mEq/L); fractional sodium excretion (FENa; %); serum potassium concentration (SK; mEq/L); urinary potassium concentration (SK; mEq/L); fractional potassium excretion (FEK; %); plasma renin activity (PRA; ηg/mL/hour); and renal hypertrophy. Statistical differences are * *p* < 0.05 vs. sham, ^§^ *p* < 0.05 vs. basal CKD, ^#^ *p* < 0.05 vs. CKD, ^Φ^ *p* < 0.05 vs. CKD LOS, and ^†^ *p* < 0.05 vs. CKD EV.

	Sham 30d (N = 20)	Basal CKD (N = 15)	CKD (N = 22)	CKD LOS (N = 22)	CKD EV (N = 15)	CKD LOS + EV (N = 15)
**BW** (g)	370 ± 10	272 ± 10 *	307 ± 8 *	306 ± 8 *	293 ± 11 *	295 ± 7 *
**UV** (mL)	22 ± 2	41 ± 3 *	45 ± 2 *	39 ± 3 *	39 ± 4 *	28 ± 2 ^§#Φ^
**SCr** (mg/dL)	0.51 ± 0.04	1.02 ± 0.13 *	0.94 ± 0.07 *	0.88 ± 0.09 *	0.92 ± 0.07 *	0.79 ± 0.05
**UCr** (mg/dL)	63 ± 5	18 ± 2 *	24 ± 2 *	31 ± 3 *	25 ± 2 *	32 ± 3 *
**CrCl** (mg/min/RBSA)	58 ± 9	17 ± 4 *	25 ± 3 *	33 ± 5 *	21 ± 3 *	23 ± 3 *
**SUr** (mg/dL)	47 ± 2	129 ± 21 *	102 ± 7 *	92 ± 4 *§	105 ± 5 *	91 ± 4 *^§^
**SNa** (mEq/L)	133 ± 1	131 ± 0	133 ± 1	134 ± 1	136 ± 1	134 ± 1
**UNa** (mEq/L)	72 ± 6	32 ± 7 *	37 ± 3 *	42 ± 3 *	42 ± 3 *	51 ± 4 *
**FENa** (%)	0.40 ± 0.05	0.89 ± 0.28	1.14 ± 0.2 *	0.94 ± 0.20	1.21 ± 0.14 *	1.04 ± 0.14 *
**SK** (mEq/L)	4.1 ± 0.1	4.1 ± 0.1	4.4 ± 0.1	4.5 ± 0.1	4.6 ± 0.2	4.9 ± 0.1 *^§^
**UK** (mEq/L)	157 ± 8	85 ± 14 *	90 ± 5 *	114 ± 6 *	94 ± 7 *	127 ± 6 *^#†^
**FEK** (%)	29 ± 3	81 ± 32	82 ± 11 *	71 ± 10 *	77 ± 5 *	71 ± 8 *
**PRA** (ng/mL/hour)	0.18 ± 0.07	0.10 ± 0.00	0.2 ± 0.0	0.24 ± 0.07	0.15 ± 0.03	0.28 ± 0.06
**Renal Hypertrophy**	3.6 ± 0.1	5.2 ± 0.2 *	5.6 ± 0.2 *	5.4 ± 0.2 *	4.7 ± 0.4 *	5.2 ± 0.2 *

## Data Availability

All data generated in the present study are included in this published article and Supplementary Materials. Further methodology details and rough data tables are fully available on request from the corresponding author.

## Supplementary Materials

The following supporting information can be downloaded at: https://www.mdpi.com/article/10.3390/cells14060434/s1, Table S1: EV total protein content determination; Table S2: Genes and primer sequences; Figure S1: Illustrative chart showing the workflow for EV obtaining and isolation; Figure S2: Experimental Protocol; Figure S3: Glomerular area occupied by ZO-1.
