# Supplementary material for: Extracellular Vesicles (EVs) Derived from Mesenchymal Stem Cells (MSCs) as Adjuvants in the Treatment of Chronic Kidney Disease (CKD)
Source: Cells. 2025 Mar 14;14(6):434. doi: 10.3390/cells14060434 (PMC11941753; doi:10.3390/cells14060434)
Supplement: Supplementary file 1 [file cells-14-00434-s001.zip › cells-3368243-supplementary.pdf]

cells

MDPI

Supplementary Material

EXTRACELLULAR VESICLES (EV) DERIVED FROM MESENCHYMAL STEM CELLS (MSC) AS ADJUVANTS IN THE TREATMENT OF CHRONIC KIDNEY DISEASE (CKD)

Supplementary Material

Supplementary Table 1: EV total protein content determination. The total protein content of obtained EV pellets was determined by a colorimetric technique (Pierce™ BCA Protein Assay Kit #23227, Thermo-Fisher Scientific), after standard RIPA protein extraction protocol. Total protein dosage was performed in 5 different samples of EV inoculums and the average protein content was calculated.

EV TOTAL PROTEIN CONTENT DETERMINATION		
Sample	Total Protein Concentration (μg/mL)	Total Protein Content (μg/inoculum)
**1**	727	109
**2**	1200	180
**3**	733	110
**4**	1079	162
**5**	1034	155
Mean		**143**

Supplementary Table 2: Genes and primer sequences. The housekeeping beta-actin (*Bact*) gene was used as an endogenous control of the PCR reaction.

GENE	Primer FORWARD	Primer REVERSE
Bact	AGGGAAATCGTGCGTGACAT	CCATACCCAGGAAGGAAGGC
Il1	ACTGAACTTCGGGTGATCG	GCTTGGTGGTTTGCTACGAC
Il2	CCAAGCAGGCCACAGAATTG	CAAATCCAACACACGCTGCA
Il4	GGAGAACGAGCTCATCTGCA	GGTGCAGCTTCTCAGTGAGT
Il6	CCATCTGCCCTTCAGGAACA	ACTGGCTGGAAGTCTCTTGC
Il10	GCTCAGCACTGCTATGTTGC	TGTTGTCCAGTCGGTCCTTC

Supplementary Figure 1

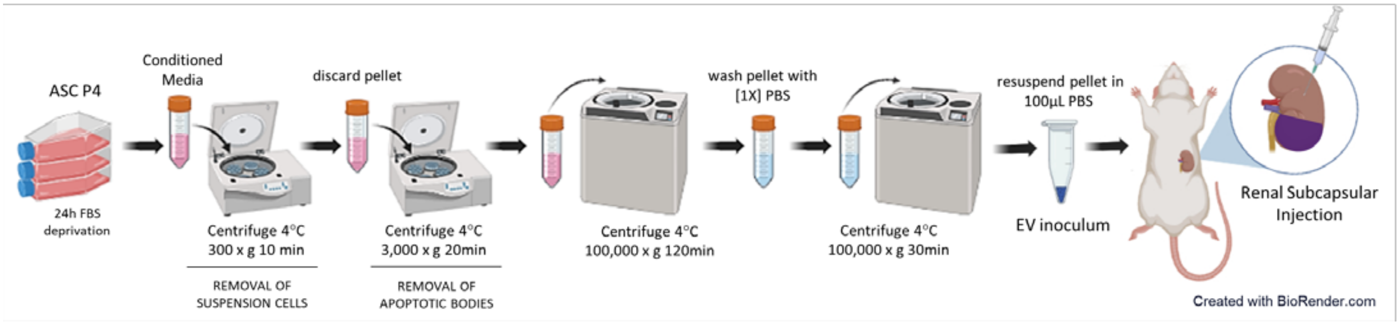

Supplementary Figure 1: Illustrative chart showing the workflow for EV obtaining and isolation. Created with BioRender.com

Supplementary Figure 2

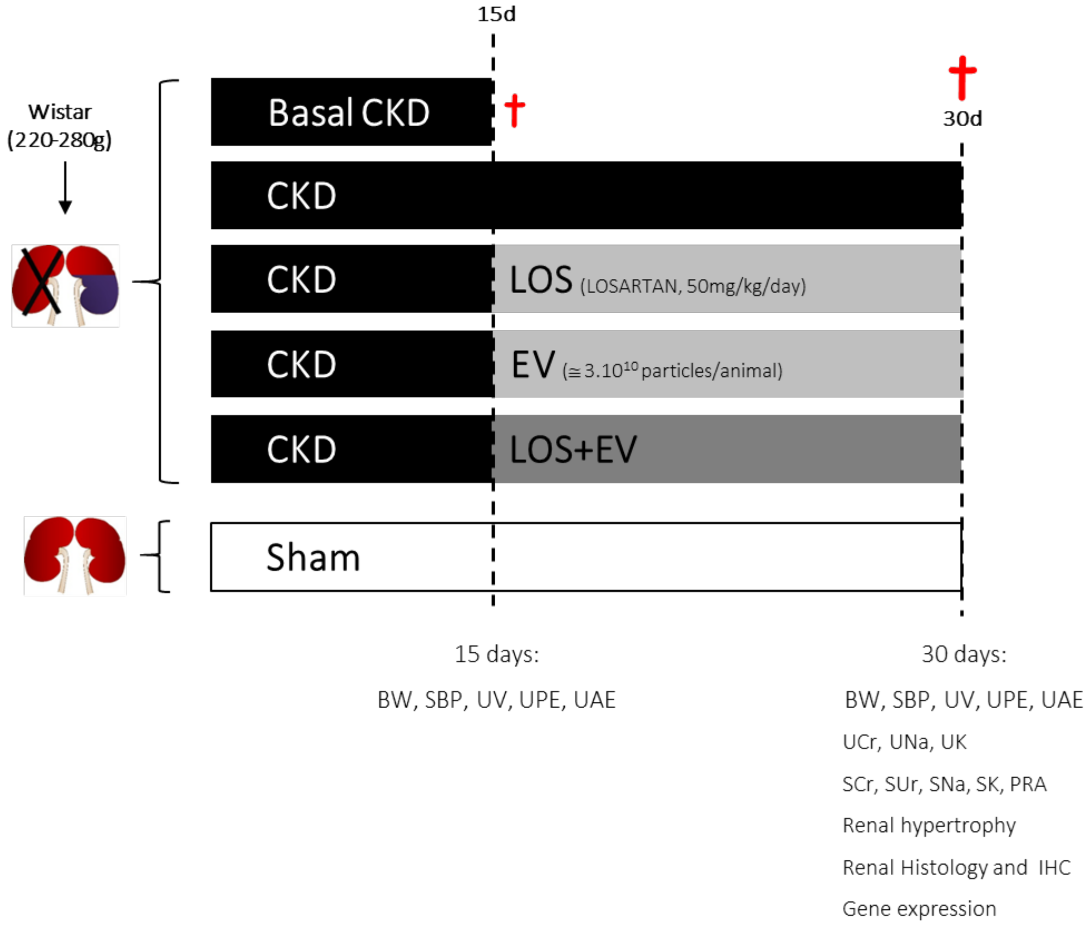

Supplementary Figure 2: Experimental Protocol. After 15 days of renal ablation, CKD animals were randomized according to their basal SBP and UPE, into five experimental groups: basal CKD (N=15), euthanized 15 days after the CKD induction; CKD (N=22), kept untreated until the 30th day after renal ablation; CKD LOS (N=22), that received 50 mg/kg/day of Losartan, diluted in drinking water, from the 15th to the 30th day after CKD induction; CKD EV (N=15), that receive a subcapsular injection of approximately 3x1010 particles of EV, after 15 days of renal ablation and followed for more 15 days of protocol; CKD LOS+EV (N=15), that receive both EV subcapsular injection and oral treatment with Losartan, until the 30th day of protocol, when they were euthanized; Sham animals (N=20) were used as control.

Supplementary Figure 3

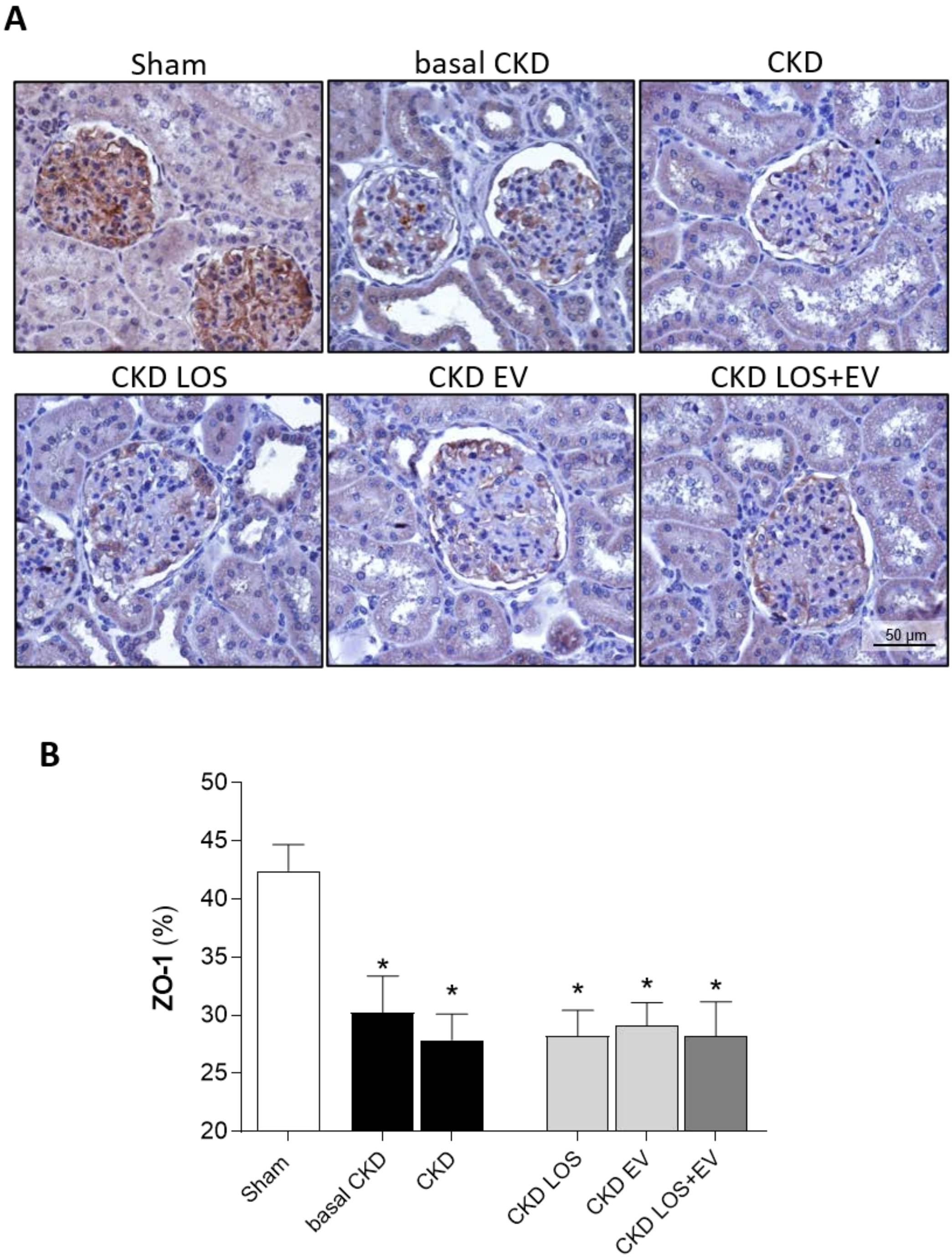

Supplementary Figure 3: Glomerular area occupied by ZO-1. (**A**) Illustrative microphotographs of immunohistochemistry for ZO-1 detection (brown staining in the glomeruli) in renal samples of animals of each experimental group, under final 400x magnification. (**B**) Bar graphs showing the percentage glomerular area occupied by ZO-1 in the different groups, by the end of the protocol. Statistical differences are: *$p<0.05$ vs. Sham, §$p<0.05$ vs. basal CKD, #$p<0.05$ vs. CKD, Φ$p<0.05$ vs. CKD LOS and †$p<0.05$ vs. CKD EV.
